# A dataset of eye gaze images for calibration-free eye tracking augmented reality headset

**DOI:** 10.1038/s41597-022-01200-0

**Published:** 2022-03-29

**Authors:** Zihan Yan, Yue Wu, Yifei Shan, Wenqian Chen, Xiangdong Li

**Affiliations:** College of Computer Science and Technology, Zhejiang University, Hangzhou, 310027 P. R. China

**Keywords:** Visual system, Saccades, Biomedical engineering

## Abstract

Eye tracking is a widely used technique. To enhance eye gaze estimation in different contexts, many eye tracking datasets have been proposed. However, these datasets depend on calibrations in data capture and its applications. We seek to construct a dataset that enables the design of a calibration-free eye tracking device irrespective of users and scenes. To reach this goal, we present ARGaze, a dataset with 1,321,968 pairs of eye gaze images at 32 × 32 pixel resolution and 50 corresponding videos of world views based on a replicable augmented reality headset. The dataset was captured from 25 participants who completed eye gaze tasks for 30 min in both real-world and augmented reality scenes. To validate the dataset, we compared it against state-of-the-art eye gaze datasets in terms of effectiveness and accuracy and report that the ARGaze dataset achieved record low gaze estimation error by 3.70 degrees on average and 1.56 degrees on specific participants without calibrations to the two scenes. Implications for generalising the use of the dataset are discussed.

## Abstract (summary)

**Table:** 

Measurement(s)	Eye Movement Measurement
Technology Type(s)	eye tracking device
Factor Type(s)	Target Movement
Sample Characteristic - Organism	Homo sapiens

## Background & Summary

Eye tracking has long been regarded as a useful technique^1^ that matches eye movements with corresponding world views and reveals the user’s real-time visual attention status and how it changes by measuring characteristics such as eye gazes^2^. Despite increasing diversity and sample size, the current eye tracking datasets mostly involve troublesome calibrations with different users and scenes. Calibration refers to the initialisation of coordinate mapping between the user’s eye gazes and viewed images^3^, thus establishing geometrical relationships between the user’s pupil movements and world views^4^. As the calibration requires strict alignment of the user’s pupil images and world views, it is vulnerable to “calibration slip” issues that are caused by head movements and headset displacements^5,6^. Thus, when a user starts to use an eye tracking device or displaces the head device during use, calibration (or recalibration) is necessary.

The existing eye gaze datasets have supplied rich resources for eye gaze estimation but are insufficient to develop calibration-free eye tracking devices that are user- and scene-independent. There are many datasets that capture images from multiple scenes. However, the images in these datasets are loosely coupled (e.g., the images extracted from the real-world scene cannot be directly used with the augmented reality headset, and the TEyeD dataset captures considerable eye gaze images from multiple scenes, but a comprehensive assessment of the dataset is still needed before use). Additionally, recent datasets focus on automatic gaze estimation^7^ and are less concerned with how to fuse multiscene eye gaze images (and their temporal and spatial features) to shape an integral dataset that enables calibration-free eye tracking.

Preparing a qualified dataset for calibration-free eye tracking is challenging because such a dataset needs to be adequately representative^8^. Being representative implies that: (a) the dataset is sufficiently large to provide diverse image samples with essential features; (b) the dataset is sufficiently small to be efficiently stored, transmitted, and processed by mainstream hardware devices (e.g., mobile devices) and algorithmic models; (c) the dataset images are transferable to alternative formats and structures;^9^ and (d) a hardware system can be easily replicated to generalise calibration-free eye tracking. Additionally, constructing such a dataset involves overcoming technical difficulties, e.g., the simultaneous presence of augmented reality displays and real-world views^10^.

We present ARGaze, a dataset of 1,321,968 pairs of eye gaze images at 32 × 32 pixel low resolution and 50 corresponding videos of world views based on a replicable augmented reality headset. The dataset was derived from 25 participants who completed a set of eye gaze tasks in both the real-world and in augmented reality. In contrast with the existing eye tracking datasets, the ARGaze dataset has several advantages. (a) It comprises a sufficient number of low-resolution images with essential eye gaze features, together with the replicable augmented reality headset, to enable the design of a calibration-free eye tracking device. Specifically, it has an extraordinary number of eye gaze images (658,168 pairs) in the augmented reality scene. To the best of the authors’ knowledge, it is by far the largest dataset with real-world and augmented reality-combined multiview and multiscene eye gaze images. (b) It is simultaneously compatible with real-world and augmented reality scenes with validated effectiveness and accuracy across scenes, and it has proven generalisability for various machine learning models (e.g., the existing invisible model and other models) for accurate calibration-free eye tracking. Importantly, the ARGaze dataset is presented together with the detailed configurations of the augmented reality headset, which helps researchers replicate the design of the calibration-free eye tracking device.

Our main contributions are twofold. First, we present the ARGaze dataset, which specialises in the design of calibration-free eye tracking devices in real-world and augmented reality scenes. The dataset has several proven advantages over the existing datasets. Second, we provide validated details, e.g., the machine learning model and hardware configurations, to allow researchers to replicate our work and effectively eliminate eye tracking calibrations before and during use. The ARGaze dataset together with the model and device outperformed the state-of-the-art eye gaze datasets in terms of the accuracy of calibration-free eye tracking across real-world and augmented reality scenes. Specifically, without any prestudy model training or calibrations, the individual participant-based eye gaze estimation error of the ARGaze model is 1.5 degrees.

A schematic overview of the study and assays is shown in Table 1.

**Table 1:** Schematic overview of the study and assay design.

Step	Description	Procedures
Data acquisition	Capture images of left eye, right eye, and world view	• Record eye images and corresponding world view images in the real-world and augmented reality scenes
Data processing	Locate and annotate markers in the real-world and augmented reality scenes, and remove unqualified images	• Identify eye areas • Remove eye blink images • Transform video footages into image sequences • Extract marker coordinates from the images
Data verification	Propose the models with which to validate the dataset in terms of eye gaze estimation (without calibration) effectiveness and accuracy	• Assemble the dataset with eye images and related coordinates • Validate the dataset with the SIFTNet- and ALSTM-FCN- hybrid model

## Methods

The acquisition of the ARGaze dataset is completed in three main steps: (a) set up experiment apparatus and environment, (b) record the images of the participants’ left and right eye and corresponding views in the real-world and augmented reality scenes during the experimental tasks, and (c) format and assemble all images of each participant and scene and import these data into the dataset. The procedural steps are summarised in Table 1. The overall dataset preparation was conducted with approval of the ethical committee of the university (see the Ethics section statement). We describe relevant details in the following sections.

### Ethics statement

The study procedures complied with the university’s research ethics regulations (ref no. CS202007). The authors were instructed to perform all procedures in accordance with the approved guidelines, including but not limited to obtaining informed consent forms from each participant before conducting any experimental measurements and keeping all private information (e.g., images of faces) anonymised. It was stated in the consent form that the participants could quit the study at any time if they felt uncomfortable or inappropriate, and the participants could withdraw all personal information.

### Participants

We recruited 30 participants through flyers, emails, websites, and social networks at the university, and each participant was paid $15. Qualified participants included undergraduate or postgraduate university students without myopia (i.e., they must have normal or correct-to-normal eyesight, not wear glasses but may have contact lenses, and have their eyesight doublechecked before the experiment) or physical disabilities, males and females in equal proportions, and individuals with good knowledge of eye tracking preferred of any age and from any background. Five participants encountered technical issues during the experiments; thus, their data were removed from the final dataset (25 participants remained). The demographics of the participants are summarised in Table 2, and the characteristics of the participants’ eyes are summarised in Table 3. The participant backgrounds and their eye features were carefully selected to ensure data diversity.

**Table 2:** Participant demography.

Gender	Age	Visual acuity on average (LogMAR)	Backgrounds
13 females, 12 males	M = 22, SD = 2.53	left eyes (M = 0.22, SD = 0.34) right eyes (M = 0.23, SD = 0.324) Note: 10 out of 25 participants had myopia and wore contact lenses during the experiments.	9 computer engineering 6 computer science 2 fine art 5 social science 3 medicine

**Table 3:** Characteristics of the participants’ eyes.

Feature Name	Values
Iris Colour (under IR-enabled camera)	7 dark, 6 medium, 12 bright
Single or double eyelids	10 single-eyelid, 15 double-eyelid
Eye shape	4 round, 21 almond; 2 protruding, 23 monolid; 5 upturned, 20 downturned

### Apparatus

#### Augmented reality headset design

**Fig. 1:**
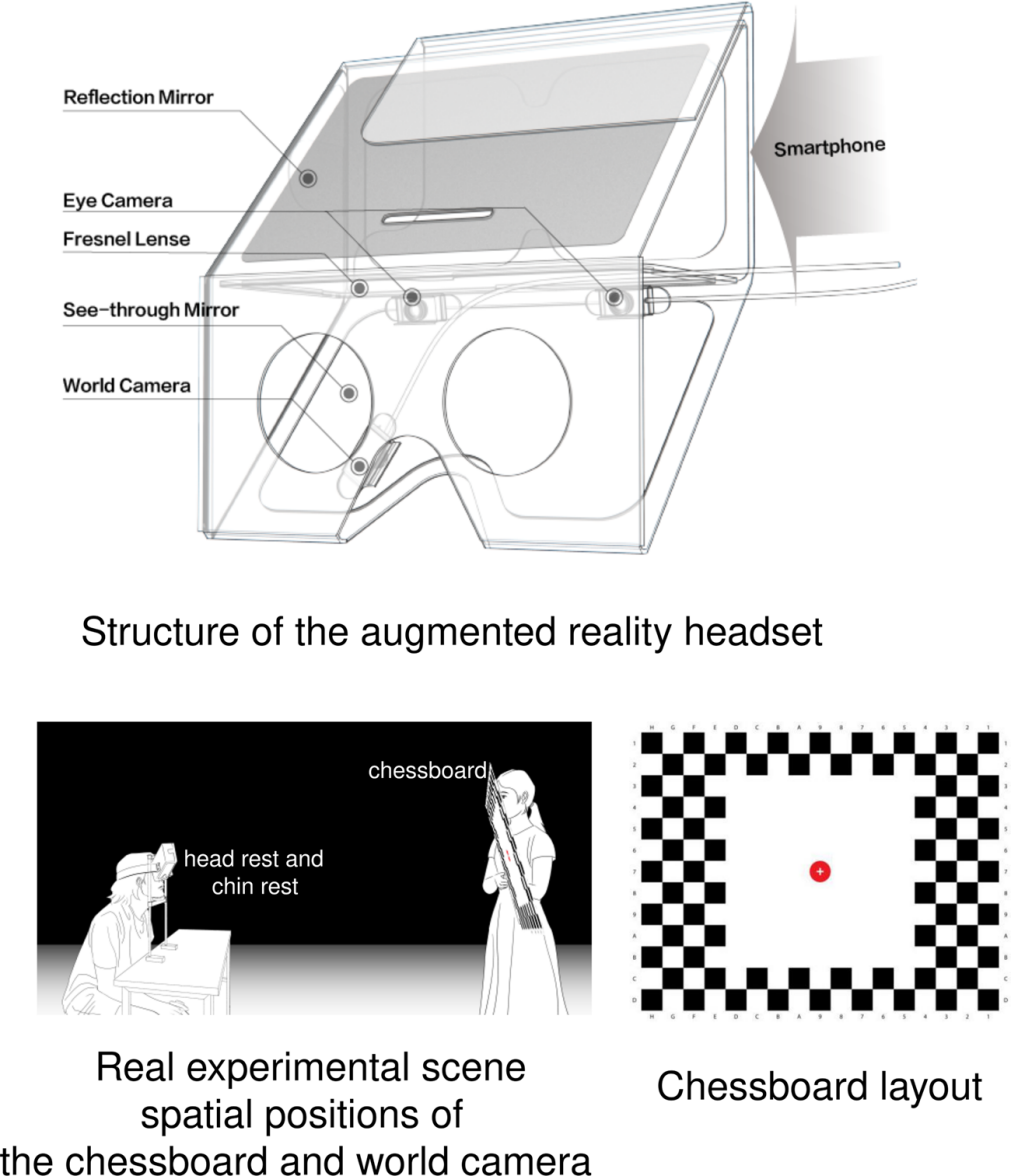
Devices and scenarios.

The eye tracking augmented reality headset was built based on the Holokit (Fig. 1 top), a cardboard augmented reality headset^11^. The Holokit headset is low-cost ($36), lightweight (407 grams), easy to configure, and with sufficient interior space to mount multiple video cameras. We attached adjustable straps to the headset to support stability while wearing the headset. The headset body consisted of a reflection mirror, Fresnel lenses, and a see-through mirror that allowed the participants to simultaneously see the real-world and augmented reality views. It used ARKit- and ARCore-compatible mobile phones to display interactive contents.

**Fig. 2:**
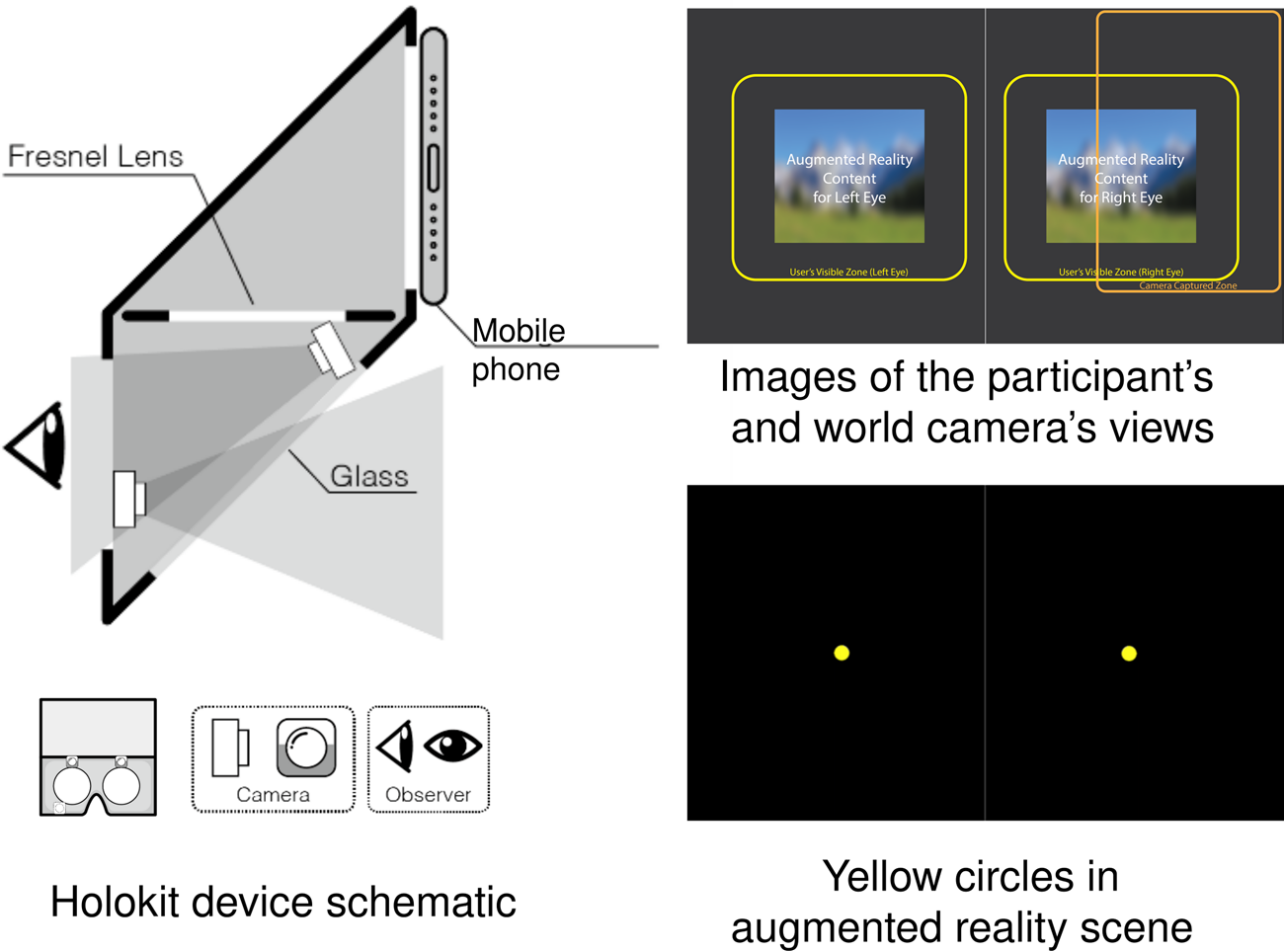
Fixtures and views.

Three infrared cameras were mounted inside the headset (all with 1080 × 720 pixel displays, 60 fps, 60 degree field of view, 20 × 10 × 5 mm size, 1/6 CSP Chip, and back focal length of 2.8 mm). Each camera had four integral infrared LEDs to illuminate pupil contours and corneal reflection. Of these cameras, the world camera was fixed at the eye side to face the see-through mirror such that it captured both augmented reality and real-world views, and the two other eye cameras were mounted opposite the eyes to capture binocular eye images. The headset has 76 degrees of field of view for augmented reality displays, but the real-world camera captures only a part of the augmented reality display because of view angle differences (Fig. 2 top right). All three cameras were connected to the host workstation computer via USB cables (6-core 2.60 GHz i7–9860H CPU, 64 G memory, Nvidia RTX 3000 GPU).

We fitted the Google Pixel3 mobile phone (4 G memory, Qualcomm 1.6 GHz quad-core CPU, Android 9) within the headset to display the augmented reality contents. Additionally, we developed head and chin rests to help the participants maintain their head position during the experiments. Importantly, the rest circumvented the “calibration slip” issue by guiding the participants’ viewing areas during free head movements (Fig. 1 bottom left).

To minimise the adverse influence of severe eye fatigue and cognitive distractions that might offset real eye gaze activities, we gave the participants a handheld game console controller, which indicates when the participants need a break. The controller was connected to the mobile phone via Bluetooth throughout the experiments. The controller was also used by the experimenter to switch between augmented reality and real-world scenes, so the participants could see different displays.

The experimental space consisted of a table (120 cm height) that held the head rest, chin rest, and headset device. The surrounding space around the table was decorated with black curtains, which provided a clear and consistent experimental environment. This also eliminated unnecessary colour reflections and distractions from ambient lighting and artefacts.

We printed an A3-sized chessboard with 3 × 3 cm white and black squares and a red circle (3 cm diameter) at the centre (Fig. 1 bottom right). The centre of the red circle was marked as the target that the participants needed to stare at, and the surrounding squares were designed to help identify the red circle’s position. To minimise distractions from board grids, we removed the grids surrounding the red circle. The chessboard was held by the experimenter and placed in front of the participants at a distance of 300 cm.

**Fig. 3:**
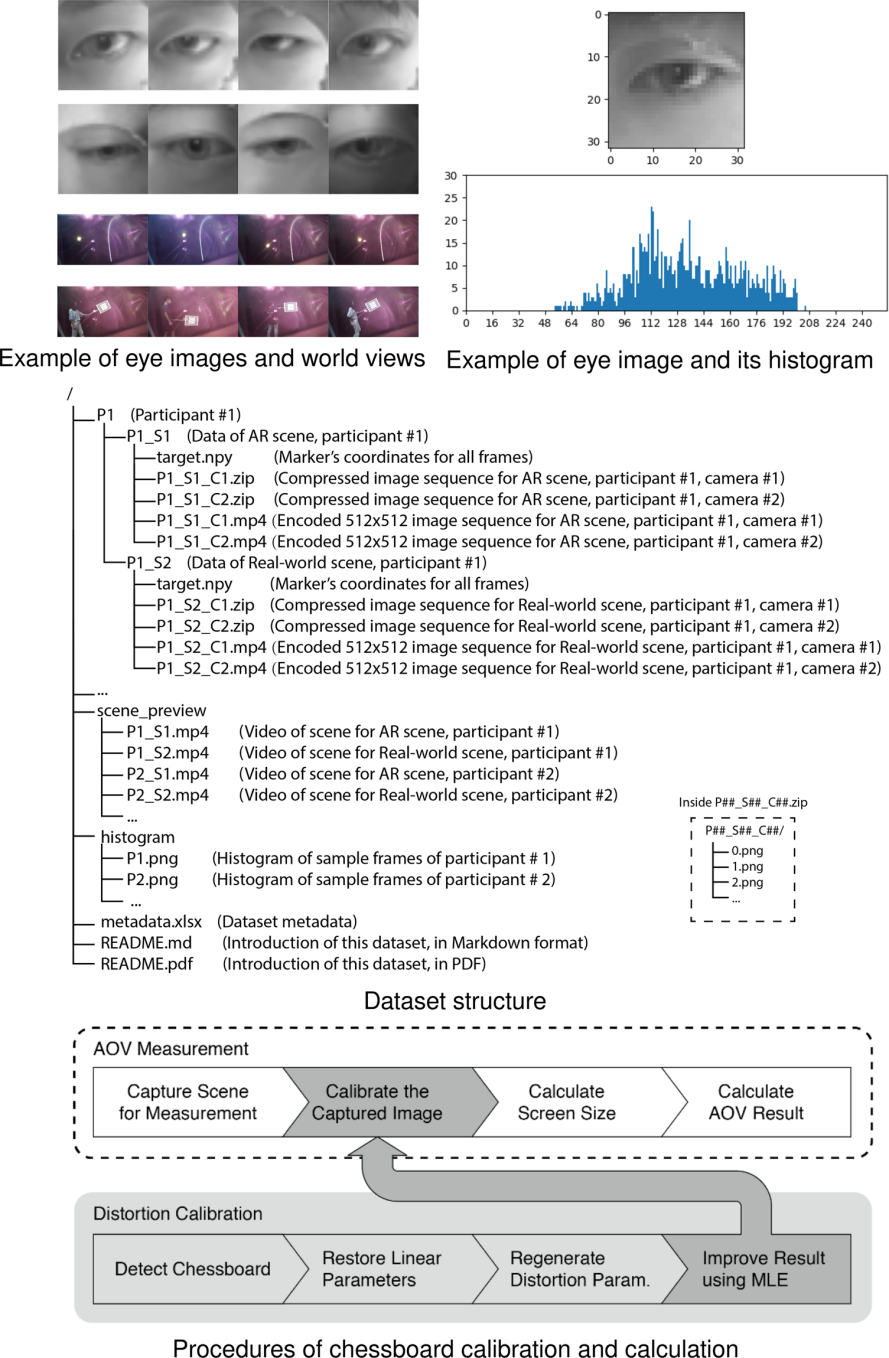
Dataset structure and procedures of the chessboard calibration and calculation.

We calculated the positions of the white and black grids to transform between the world coordinate and camera coordinate systems. The method of calculation was already proven by early research^12^. The calculation results were used to reconstruct the world camera’s distortion parameters and angles of view (AOV), and the mapping between screen coordinates (in pixels) and gaze directions (in degrees) was achieved. The positions between the chessboard and world camera are depicted in Fig. 1. After a distortion calibration, we positioned the chessboard 200 cm away from the world camera at different angles and obtained the AOV (*α*,*β*) with the following equations. The equations described the mapping from screen-space coordinates to world-space directions and transformed screen coordinates into angles of eye gaze direction.

1 $$\left\{\begin{array}{c}u=arctan\left(\frac{x-{x}_{0}}{M}tan\alpha \right)\\ v=arctan\left(\frac{y-{y}_{0}}{N}tan\beta \right)\end{array},\right.$$

where (*x*, *y*) are the screen-space coordinates and $\left({x}_{0},{y}_{0}\right)$ are the centre coordinates of the captured image. According to early research^12^, (*α*, *β*) refers to the angle of view in the horizontal and vertical directions; (*M*, *N*) refers to the horizontal and vertical screen resolutions; and $\left(u,v\right)$ is the final output, which refers to the angular offset direction in angles related to the main (forward) axis of the camera (horizontal and vertical, respectively). Given (${x}_{0},{y}_{0},M,N$), we estimated AVO and gave pairs of (*x*, *y*) and (*u*, *v*) by sampling the black and white chessboard corners and solved for *α* and *β*. After parameter (${x}_{0},{y}_{0},M,N,\alpha ,\beta$) validation, we mapped the coordinates into corresponding directions and estimated the angular error with the mapping. The main flows of distortion calibration and AOV calculation are shown in Fig. 3.

#### Software design

Running on the mobile phone and the workstation was software for displaying augmented reality contents and recording eye gaze images and world views, respectively. The software in the workstation was a multistream video recording application called OBS studio^13^. The software was responsible for monitoring real-time video streams (two eye gazes and one world view) and recording all video footages in Matroska Videos (MKV) format in individual folders named by participant orders and task types (see image examples in Fig. 3). The OBS Studio automatically transformed video footages into MPEG-4 format. In addition, a clock was displayed on the video monitoring window to indicate the current length of recording.

The mobile phone application was built with ARCore and Unity3D. It consisted of two binocular windows, each showing an identical randomly moving yellow circle (Fig. 2). The yellow circle’s size and moving velocity were justified such that: (a) the circle is large enough to be visually noticeable and small enough to circumvent unexpected distraction, and (b) the circle’s movements are visually traceable by the participants. Additionally, the yellow circle’s movements were randomised. Algorithms of circle movement control are described below.

We set P(t) as the main function controlling circle movements, where *t* is linearly correlated with the current time and *α* and *ε* are the coefficients of normalisation function ${N}_{\beta ,\varepsilon }\left(x\right)$ that indicate the size of the graph and the smoothness around the edge, and calculated $\beta =\alpha -\varepsilon$. We set $\varepsilon =0.05$, $\alpha =1.0$ on the y axis, and $\alpha =0.8$ on the x axis, leaving us with:

2 $$F\left(t\right)=\left\{\begin{array}{c}r=1.52cos\left(5.7t\right)\\ \theta =t\end{array},\right.$$

where $F\left(t\right)$ is the parametric function to generate the proposed target position in polar coordinate form. R and *θ* are the distance from the origin and the angle from the x axis, respectively.

3 $${N}_{\beta ,\varepsilon }\left(x\right)=\left\{\begin{array}{c}x,-\beta \le x < \beta \\ \varepsilon ,\beta \le x < \beta +\pi \varepsilon \end{array},{N}_{\beta ,\varepsilon }\left(x\right)=-{N}_{\beta ,\varepsilon }\left(x+\beta \right),\right.$$

where N(x) is the normalisation function for the Cartesian coordinate values. It is a smooth and continuous periodic function that maps the value to the $\left(-\beta ,\beta \right)$ range, which makes the target move smoothly within the boundary in a more unpredictable way, and has two constant parameters, *β* and *ε*. *β* is the range of the target position; *ε* is the degree of easing of reflection when the target is near the boundary. The greater the *ε* is, the longer the easing distance, and vice versa.

4 $$P\left(t\right)=\left\{\begin{array}{c}x={N}_{{\beta }_{x},\varepsilon }\left(rcos\left(\theta \right)\right)\\ y={N}_{{\beta }_{y},\varepsilon }\left(rsin\left(\theta \right)\right)\end{array}\right.,$$

where $P\left(t\right)$ are the Cartesian coordinates of the target display position. x and y are the horizontal and vertical components, respectively. *β*_*x*_ and *β*_*y*_ are constants, and r and *θ* are the components of the polar coordinate $F\left(t\right)$. This expression first converts polar coordinates into Cartesian coordinates and then normalises them and outputs coordinates with time as a parameter.

#### Procedures

**Fig. 4:**
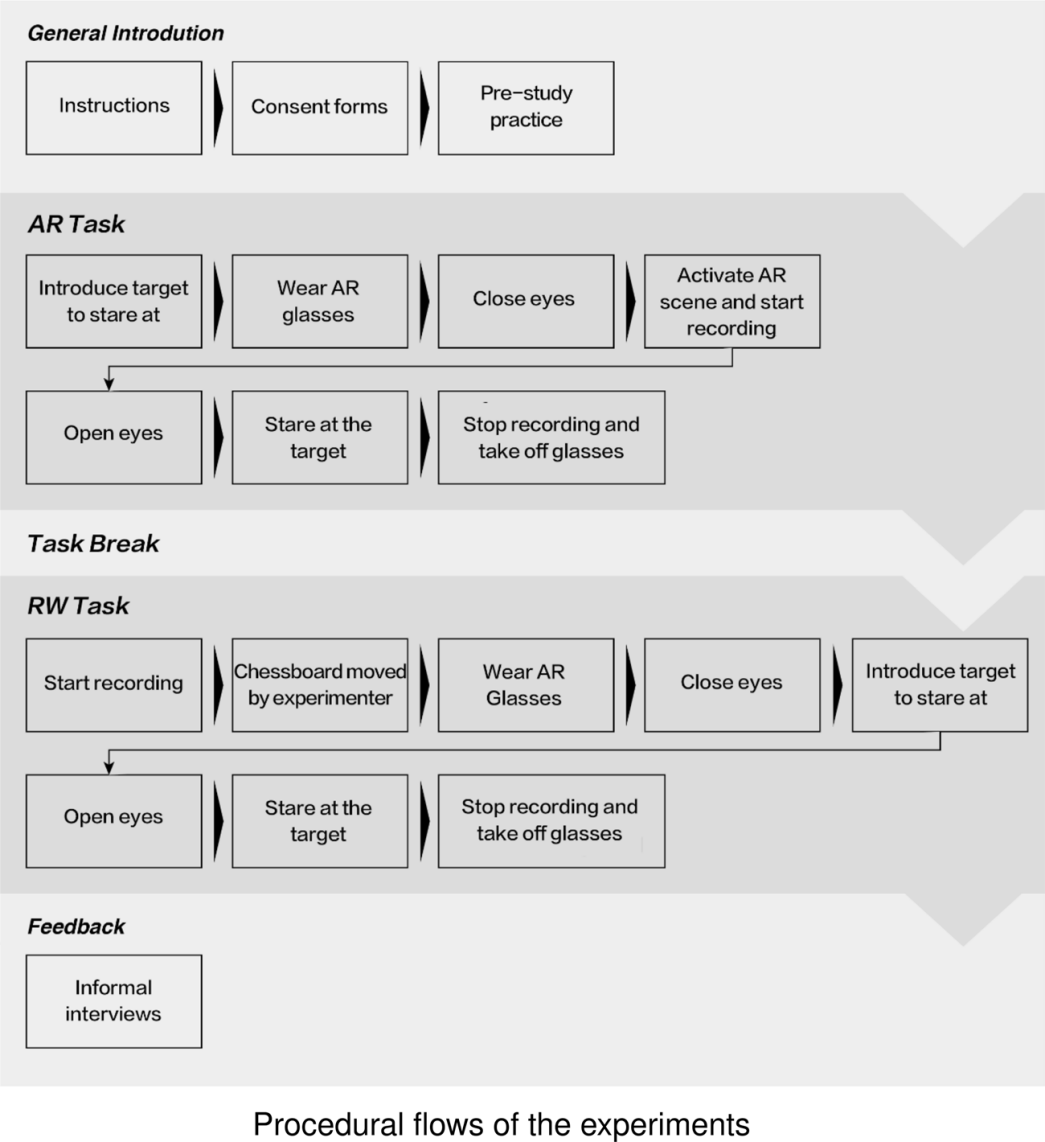
Procedural flows of the experiments.

The experiment consisted of two sequential tasks, both following the same procedural flows. The first task was in the augmented reality scene, which required the participants to continuously stare at the yellow moving circle for approximately 10 min. The second task was in the real-world scene, which required the participants to continuously stare at the red circle of the moving chessboard for approximately 10 min. There was a 5 min break between the two task sessions, and the participants could relax their eyes (these data were removed). The overall experimental procedures are shown in Fig. 4.

The experiments started with participant preparation, in which the experimenters introduced experimental objectives and procedures, confirmed the participants’ understanding of the experimental apparatus and procedures, and checked their eyesight. The introduction included instructions for how to use the headset and game console controller, respond to eye fatigue and adjust body gestures as well as targets to stare at. Sequences of the two tasks and related requirements for visual attention were explained to the participants. Guidelines for facilitating low visual attention status and keeping the headset where it was positioned were explained. The participants’ understanding of these requirements was validated with semiformal interviews by the experimenters.

Following the introduction and confirmation, participants’ eyesight was checked. In the augmented reality scene, the participants were asked to trial the headset and check whether there were any “ghost shadows” in the yellow circle. Ghost shadows refer to multiple overlapping images of the target object, which are commonly caused by astigmatism and would impair the participants’ ability to identify visual objects. In the real-world scene, the experimenters checked whether the participants could see the chessboard clearly from 200 cm away within the experimental space. This was to identify the space where the chessboard remained visible to the participants. With the experimenters’ assistance, the participants put on the headset and started a 30 s practice. During the practice, the participants were asked to “think aloud” about their headset use, so the experimenters could continue to optimise its comfort. The order of the experimental scenes was randomised.

The experimental procedures of the AR scene were as follows. First, the participants found a comfortable position of the head with the chin rest and wore the eye tracking headset. This process was facilitated by the experimenters, who subsequently adjusted mobile phone positions and checked image capture. Second, the experimenters started video recording software in the workstation and activated the augmented reality application in the mobile phone. Formal experimental tasks were initiated from this time and terminated when the participants had effectively run the tasks for 10 min. Finally, the participants were given an informal interview about task experience.

Likewise, the experimental procedures of real-world scenes were as follows. First, the participants were instructed to sit in front of the table, which was 300 cm away from the chessboard. Second, the participants were helped by the experimenters to wear the eye tracking headset to continuously follow the red circle at the centre of the chessboard. The chessboard was moved by the experimenter. The process was terminated when the participants had effectively run the tasks for 10 min. Finally, the experimenters conducted routine informal interviews and questionnaires regarding task experience.

### Data cleaning

**Fig. 5:**
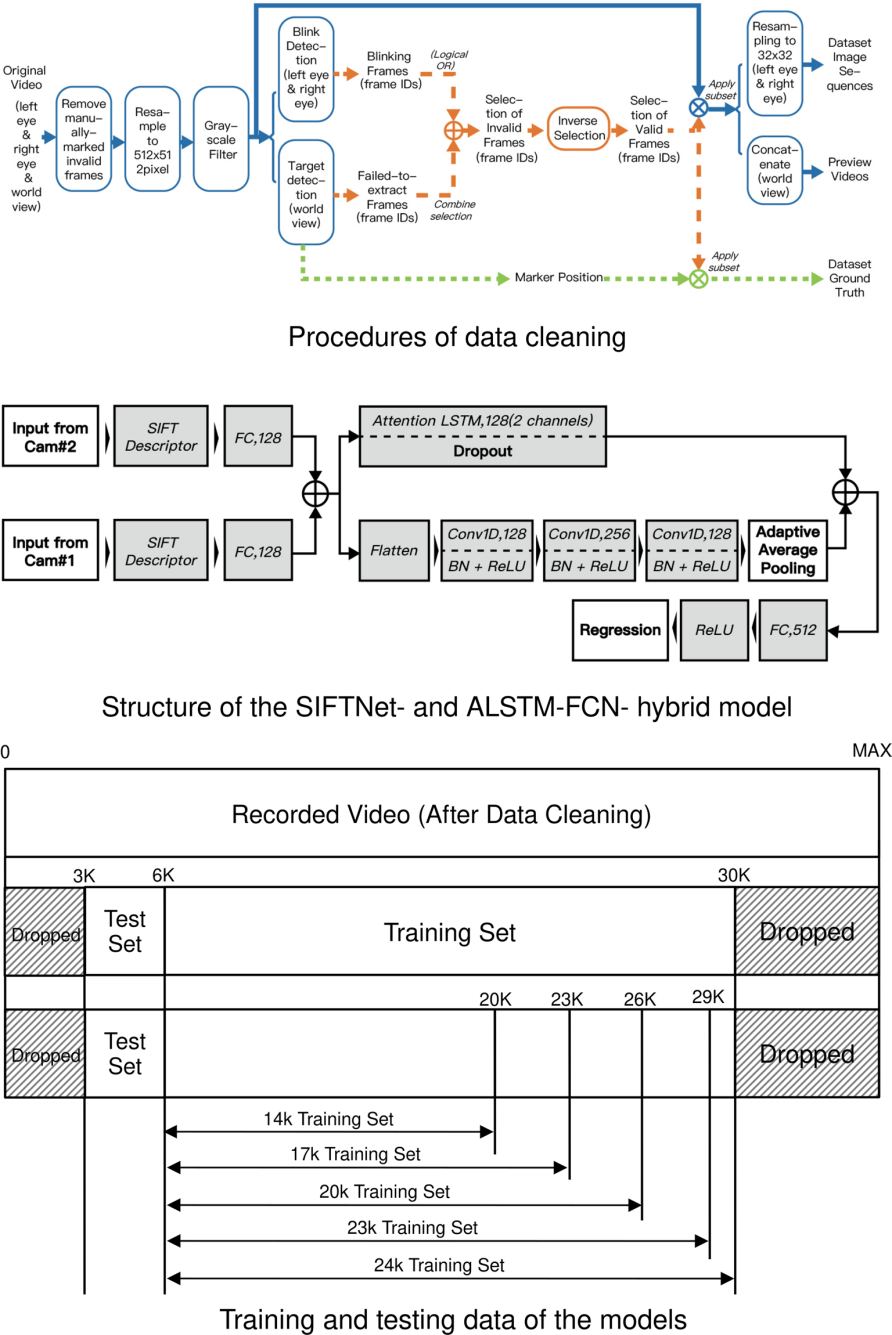
Network structure and training method of the model.

We conducted data cleaning to filter out unqualified data, such as images with closed eyes, eye blinking, and eye absence. Selected images of the world camera were combined into video footage for preview, and the images of the left and right eyes were transformed into 32 × 32 pixel image sequences as a part of the dataset. Corresponding with the selected images, a set of ground truth data of eye gazes and marker positions was supplied in the dataset. The main procedures used for data cleaning are described in Fig. 5. All code used for data cleaning and algorithms used to detect unqualified images are available at https://github.com/xdc-lab/ARGazeCodes.

The process of eye blinking detection is explained as follows. First, we synchronised the video clips of the real-world view and the left and right eyes and removed the manually marked invalid video images. The experimenter monitored the participants’ state and marked the unusable range of data during the experiment. We resized the images into 512 × 512 pixel format and transformed them into greyscale for consistent image contrast.

**Fig. 6:**
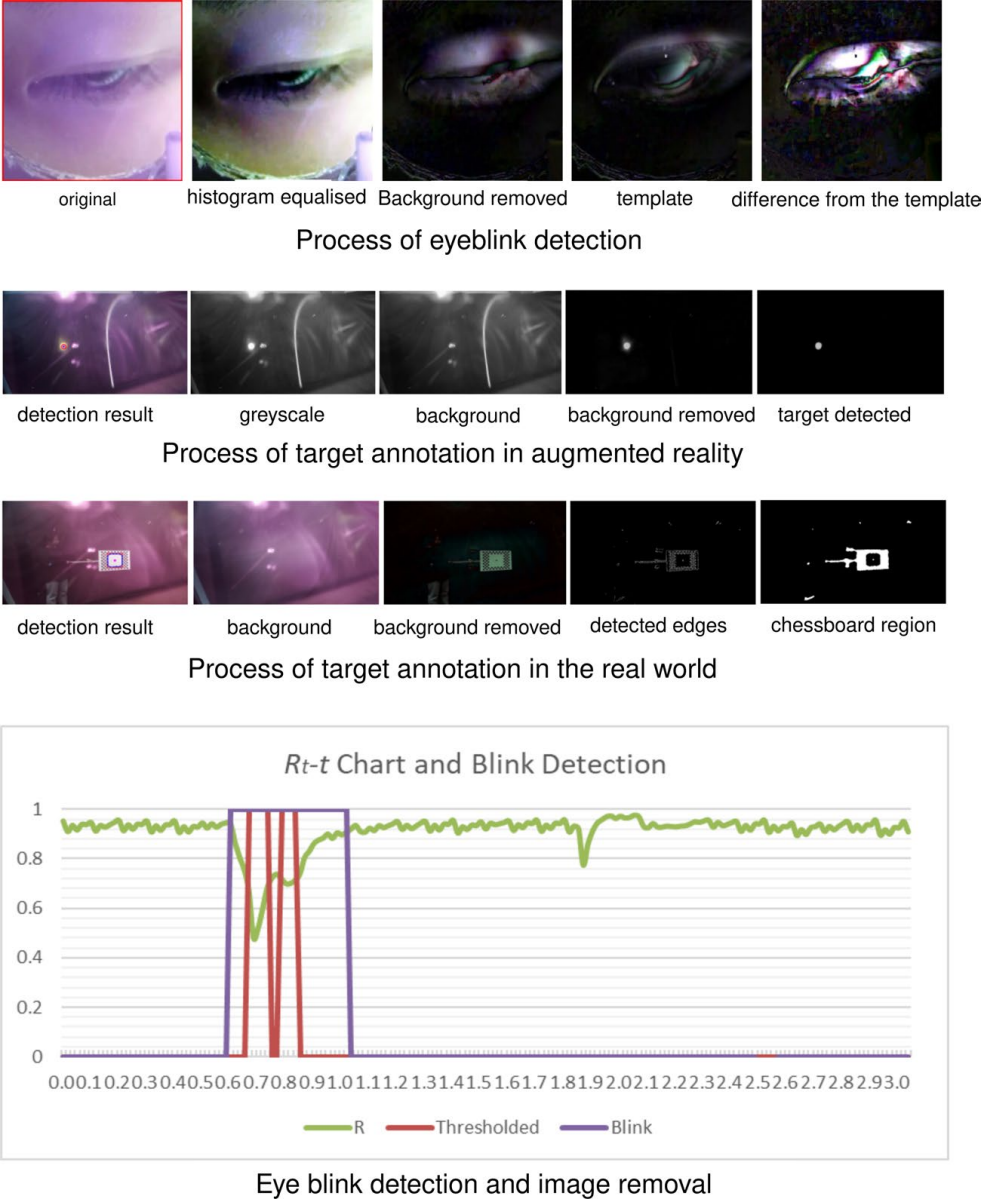
Examples of data processing.

Second, we conducted eye blinking detection to filter out images with closed eyes. The method of eye blinking detection was derived from the research of Krolak *et al*.^14^, and its effectiveness has been validated in many studies. It calculates the resemblance between the current frame (${F}_{t}$) and the standard image that was regarded as the template (${T}_{t}$) to effectively detect eye blinking. The correlation coefficient is set as ${R}_{t}=R\left({T}_{t},{F}_{t}\right)$. To eliminate false eye blinking detection that can be caused by possible eye camera position changes, we adopted a floating template, which was computed by linearly accumulating previous images. The iterative arithmetic expression that calculates the template image for each timepoint *t* is described as follows:

5 $${T}_{t+1}=\left\{\begin{array}{cc}0.4\left({F}_{t}-{T}_{t}\right)+{T}_{t} & ,\neg Blinkin{g}_{t}\\ 0.2\left({F}_{t}-{T}_{t}\right)+{T}_{t} & ,Blinkin{g}_{t}\end{array}\right.,$$

where the addition and subtraction of frames (i.e., ${F}_{t}$ and ${T}_{t}$) are defined as the independent addition and/or subtraction over all channels for all pixels. The overflowed or underflowed values are truncated to the upper bound and the under bound, respectively. An example of the process of eye blinking detection is demonstrated and the detection results are summarised in Fig. 6.

We extracted the eye blinking images by testing ${R}_{t}$ against the fixed threshold ($T{H}_{R}$).

6 $$Blinkin{g}_{t}={R}_{t}\le T{H}_{R},$$

where $Blinkin{g}_{t}$ is the Boolean value (values of true s and false s) that indicates whether the participant is blinking at time *t*.

To eliminate eye blinking images, additional video images of before and after the eye blinking event were also removed. Specifically, in Fig. 6, R indicates eye blinking events, Threshold shows eye blinking detection results, Blink shows the removal of images with potential eye blinking.

Finally, we removed the first and last 2,000 images of each participant’s data, as these data were quite likely to be affected by task starting and ending operations. We gained 26,552 pairs of images (from the left and right cameras) from each participant session (with only one exception within 50 sessions, the AR scene from Participant 2 only contained 20,920 pairs). One example of eye images and corresponding world views is demonstrated in Fig. 3.

## Data Records

The ARGaze dataset consists of 1,321,968 eye gaze images in total from 25 participants. It is now openly available in the repository of 10.17605/OSF.IO/CJXYN^15^. The images of the dataset are organised per participant and per experimental scene in hierarchical directories (see full structures of the directory in Fig. 3). In the top-level directory, there is a README.md file, which describes the details of file formats and related notes. We also provide a PDF version of this document for convenience. The directory also includes the metadata.xlsx file that records details of image size and quality. In the subdirectory histogram, there are preview images of the participants’ eyes and corresponding histograms (Fig. 3). In the subdirectory scene preview, video clips are generated from 1280 × 720 pixel image sequences of the world camera. The overall length of these video clips is shorter than the task durations because the data cleaning process has removed a number of invalid images. Specifically, the length of the video clips is approximately 7 min 23 sec (26,552 frames at approximately 60 fps, which is the original frame rate during the session) for most scenes that contain 26,552 images. The theoretical length of preview videos is recorded in the metadata.xlsx file. The eye gaze images are organised in different image directories that are named by their participant number (e.g., P1). Within each image directory, the images are divided into two directories, P1_S1, according to the experimental scenes.

Taking directory P1 as an example, there are two subdirectories, P1_S1 and P1_S2. S1 and S2 refer to augmented reality scenes and real-world scenes, respectively. In directory P1_S1, there is a .npy file that details the coordinates of eye gazes and the resampled eye gaze images are encoded in two .mp4 videos in 512 × 512 pixels, and there are two. zip files—P1_S1_C1.zip—that contain eye gaze images of the left and right eyes. The eye gaze images are.png files in 32 × 32 pixel 8-bit greyscale and are named in timestamps that can be easily synchronised with corresponding eye images and real-world views. The high-resolution version of these 32 × 32 pixel greyscale eye gaze images is also provided in an encoded.mp4 video file in the same directory.

## Technical Validation

The dataset is prepared to enable the design of a calibration-free eye tracking device. We are motivated to present the ARGaze dataset, together with the hardware configurations and machine learning model, to develop the augmented reality headset that supports user- and scene-independent eye tracking. The ARGaze dataset is particularly useful to construct an eye tracking device that no longer needs conventional calibrations with any users in either the real-world or in augmented reality. In this section, we describe details of technical validation of the proposed dataset by evaluating its effectiveness (i.e., how reliable and valid is the dataset) and generalisability (i.e., how generalising could the dataset be used in alternative applications).

### Ground truth acquisition

Before we proceed to technical validation of the dataset, we need to acquire ground truth such as the centre coordinates of the red circle of chessboard. The process added annotations of eye gaze positions to all images. The eye gaze positions are formatted in a 2D vector (*x*, *y*) in the world camera coordinate system. Unlike conventional eye tracking applications that often set the coordinate centre at the eyes (or midway between the two eyes), we established a mapping between the camera-centred coordinate system and eye-centred coordinate system. Put simply, we defined the 3D coordinate system as a Cartesian system whose origin is at the focus of the world camera and whose original position $\left({x}_{w},{y}_{w}\right)$ refers to the ray $\left(\frac{1}{{z}_{0}}\left({x}_{w}-{x}_{0}\right),\frac{1}{{z}_{0}}\left({y}_{w}-{y}_{0}\right),z\right)$ ($z\ge 0$, ${x}_{0}$, ${y}_{0}$ and ${z}_{0}$ are device-dependent constants related to the camera FOV and resolution).

We considered participant head positions to be fixed with the world camera, as the headset was tightly mounted on the participant’s head throughout the experiments. Additionally, since the participants needed to put their eyes in front of the headset, the participants’ peripheral eye areas were similarly captured. Given that, we assume that the midpoint of the participant’s eyes (i.e., the origin of the “desired” coordinate system) is $\left({x}_{1},{y}_{1},{z}_{1}\right)$, where *x*_1_, *y*_1_ and *z*_1_ are all device-dependent constants. Assuming the plane of markers is $ax+by+cz+d=0$, it is obvious that the 3D position of the marker is:

7 $$\left(\frac{1}{{z}_{0}}\left({x}_{w}-{x}_{0}\right),\frac{1}{{z}_{0}}\left({y}_{w}-{y}_{0}\right),-\frac{1}{c{z}_{0}}\left(a{x}_{w}+b{y}_{w}+\left(d{z}_{0}-a{x}_{0}-b{y}_{0}\right)\right)\right).$$

To calculate the 2D coordinates (representing a direction) that is needed for eye gaze model training and estimation, we transform the coordinate system with a desired origin. The transformed coordinate of the marker is:

8 $$\left(\frac{1}{{z}_{0}}\left({x}_{w}-{x}_{0}-{z}_{0}{x}_{1}\right),\frac{1}{{z}_{0}}\left({y}_{w}-{y}_{0}-{z}_{0}{y}_{1}\right),-\frac{1}{c{z}_{0}}\left(a{x}_{w}+b{y}_{w}+\left(d{z}_{0}-a{x}_{0}-b{y}_{0}-c{z}_{0}{z}_{1}\right)\right)\right).$$

Therefore, the transformed direction $\left({t}_{x},{t}_{y}\right)$ can be presented by projecting on plane $x=y=0$ as follows:

9 $$\left\{\begin{array}{c}{t}_{x}=\frac{{x}_{w}-{x}_{0}-{z}_{0}{x}_{1}}{-\frac{a}{c}{x}_{w}-\frac{b}{c}{y}_{w}+\left(\frac{a{x}_{0}+b{y}_{0}-d{z}_{0}}{c}+{z}_{0}{y}_{1}\right)}+{t}_{x0}\\ {t}_{y}=\frac{{y}_{w}-{y}_{0}-{z}_{0}{y}_{1}}{-\frac{a}{c}{x}_{w}-\frac{b}{c}{y}_{w}+\left(\frac{a{x}_{0}+b{y}_{0}-d{z}_{0}}{c}+{z}_{0}{y}_{1}\right)}+{t}_{y0}\end{array}.\right.$$

Given that $u=-\frac{a}{c},v=-\frac{b}{c},A=-{x}_{0}-{z}_{0}{x}_{1},B=-{y}_{0}-{z}_{0}{y}_{1},C=\frac{a{x}_{0}+b{y}_{0}-d{z}_{0}}{c}+{z}_{0}{y}_{1}$, and $u,v,A,B,C$ are device-dependent constants:

10 $$\left\{\begin{array}{c}{t}_{x}=\frac{{x}_{w}-A}{u{x}_{w}+v{y}_{w}+C}+{t}_{x0}\\ {t}_{y}=\frac{{y}_{w}-B}{u{x}_{w}+v{y}_{w}+C}+{t}_{y0}\end{array}\right..$$

Device-dependent mapping from the extracted marker position to the device-independent gaze location involves 7 parameters and can be located using at least 4 points of mapping. Given that, we can transform the origin of the camera coordinate system into the centre point of the participant’s eyes and map the 3D position back to 2D coordinates representing gaze direction.

We annotated yellow circles in the augmented reality scene (Fig. 6). To ensure annotation accuracy, we removed image backgrounds with the MoG background modelling algorithm^16^ and enhanced the images using morphological methods with a circular structuring kernel. Then, we used Hough circle detection to detect yellow circles in the images. Additionally, we used a threshold of the mean depth value of the circle to gain accurate detection.

Likewise, annotating the red circles in real-world scenes followed similar procedures as in the augmented reality scene (Fig. 6) and included background removal, canny edge detection, and blurring and thresholding with the colour images. Finally, it annotated the contours of the chessboard and calculated coordinates at the centre.

### Models for dataset validation

We developed a deep learning model—the SIFTNet- and ALSTM-FCN- hybrid model—to validate the dataset (see the model structure in Fig. 5). The model is composed of mainstream machine learning networks such as ALST and FCN, which are accessible to researchers. Additionally, we derived the state-of-the-art eye gaze estimation model from InvisibleEye research and implemented it as the benchmark of dataset validation^17^. Both models were constructed with the same training and testing data. Extraction of these data is illustrated in Fig. 5.

The model extracted features with SIFT descriptors, which is a classic shape feature expression descriptor, due to the characteristics of pupil spots and annular irises often found in eye pictures. In addition, the combination of traditional CV techniques and deep learning can leverage the computational resources required for and accuracy of edge computing, which can be widely applied in embedded devices on AR hardware. Specifically, the tensors consisting of the magnitude in the eight directions in the 4 × 4 subregions in the image were flattened into a 128-dimensional vector, which was normalised by a second norm, and values exceeding 0.2 were set to 0.2 before being normalised by the second norm again, and the output of the 128-dimensional vector was fed into the next layer.

We calculated SIFT descriptors with all images from the three cameras and constructed a fully connected layer with 128 hidden units and ReLU activation. We combined the SIFT descriptors of the two cameras into a dual-channel data stream and simultaneously passed to the temporal convolutional network and attention LSTM network. The temporal convolutional network is a fully convolutional network (FCN) stacking three temporal convolutional blocks with output channel sizes of 128, 256, and 128. Each of the blocks consists of a temporal convolutional layer (the kernel of which uses a size of 8) with batch normalisation (with a momentum of 0.1 and an epsilon of 10^−5^) and a ReLU activation function. Before passing the results to the merging layer, adaptive average pooling is applied. In the attention LSTM step, features are passed into a two-layer-stacked bidirectional LSTM network with 128 hidden states and a dropout of probability 0.8 in between. The result of the LSTM network is then taken into the attention mechanism and assigned a dropout probability of 0.8. The results of the two layers are then merged (into a single channel) and passed into a linear layer of 512 hidden cells with ReLU activation, and the final output and x and y coordinates of the gaze direction are predicted by a linear regression layer. We chose the Adagrad algorithm with a learning rate of 0.005 as the optimiser, and the mean squared error of the predicted and real coordinates as the loss function. The initial parameters of the network were randomly set.

We use the MSE of prediction and ground-truth coordinates as the loss function during training, but in the evaluation stage, we use the MAE of the distance between the ground-truth coordinate ${Y}_{0}=\left({x}_{0},{y}_{0}\right)$ and the predicted coordinate $Y=\left(x,y\right)$. The results are based on the losses in the validation process, which can be approximately converted into degrees using the calibration plate. The mathematical representations of the loss calculation are described below.

11 $${L}_{Training}=\frac{1}{N}\sum {({\parallel Y-{Y}_{0}\parallel }_{2})}^{2}=\frac{1}{N}\sum {((x-{x}_{0})}^{2}+{(y-{y}_{0})}^{2}),$$

where N is the total data volume, ${Y}_{2}$ is the second norm of *Y* indicating the distance between the true coordinates and the predicted coordinates, and ${L}_{Training}$ refers to the loss during training.

12 $${L}_{Validating}=\frac{1}{N}\sum {\parallel Y-{Y}_{0}\parallel }_{2}=\frac{1}{N}\sum \sqrt{{(x-{x}_{0})}^{2}+{(y-{y}_{0})}^{2}},$$

where ${L}_{Validating}$ is the loss incurred during validation.

### Validation results

#### resolutions of images in the dataset

High-resolution images have rich features but also introduce distractive saliencies, and processing these images is both time- and computation-consuming. In contrast, low-resolution images have insufficient features for accurate detection. Therefore, we needed to find the trade-off between image resolutions and computational efficiency before dataset validation.

The images of our intermediate dataset are in 512 × 512 pixel format. We derived five separate copies of single-channel greyscale images of the dataset at resolutions of 5 × 5, 8 × 8, 12 × 12, 16 × 16, and 32 × 32 pixels. The selection of these resolutions was grounded in early work^17^, which indicated that 5 × 5 pixel resolution had the lowest eye gaze detection error and that the accuracy was unlikely to be affected with resolutions of 32 × 32 pixels and above. We tested the eye gaze detection error of individual participants in both experimental scenes at these resolutions. The results indicate that 12 × 12 pixels is sufficient. However, we decided to provide 32 × 32 pixel images in our published dataset to balance dataset size and effectiveness.

We derived the model from InvisibleEye and trained it with the dataset at the same 5 × 5 pixel resolution. The InvisibleEye model reaches 1.79 degrees (i.e., the lowest estimation error) on the InvisibleEye dataset using images from four cameras. In contrast, the ARGaze dataset used two main cameras for eye gaze image capture. Based on the ARGaze dataset, the InvisibleEye model achieved a 1.19 degree estimation error in the augmented reality scene and a 1.39 degree estimation error in the real-world scene (both refer to the lowest estimation error).

**Fig. 7:**
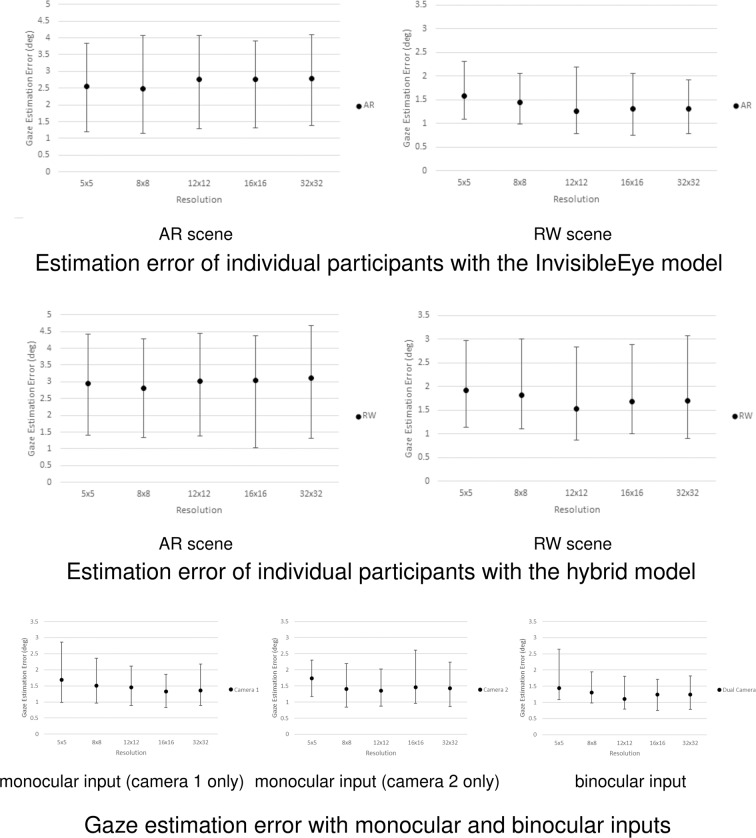
Result of estimation error with different models and inputs.

For 5 × 5 pixel images, our model can achieve an average prediction error for 1.57 degrees in scenes and 1.95 degrees in real-world scenes, which is higher than that of InvisibleEye. The average estimation error for the InvisibleEye model is 2.56 degrees in the augmented reality scene and 2.95 degrees in the real-world scene. The results showed that our hybrid model outperformed the InvisibleEye model with 71.3% lower estimation error. We evaluated the two models’ detection error using the same framework codes that were trained for the same maximum epoch (Fig. 7 first row and second row). Furthermore, the results indicate that the 32 × 32 pixel resolution is a turning point of eye gaze estimation accuracy in both real-world and augmented reality scenes, which confirms the effectiveness of the resolution.

#### numbers of images in the dataset

**Fig. 8:**
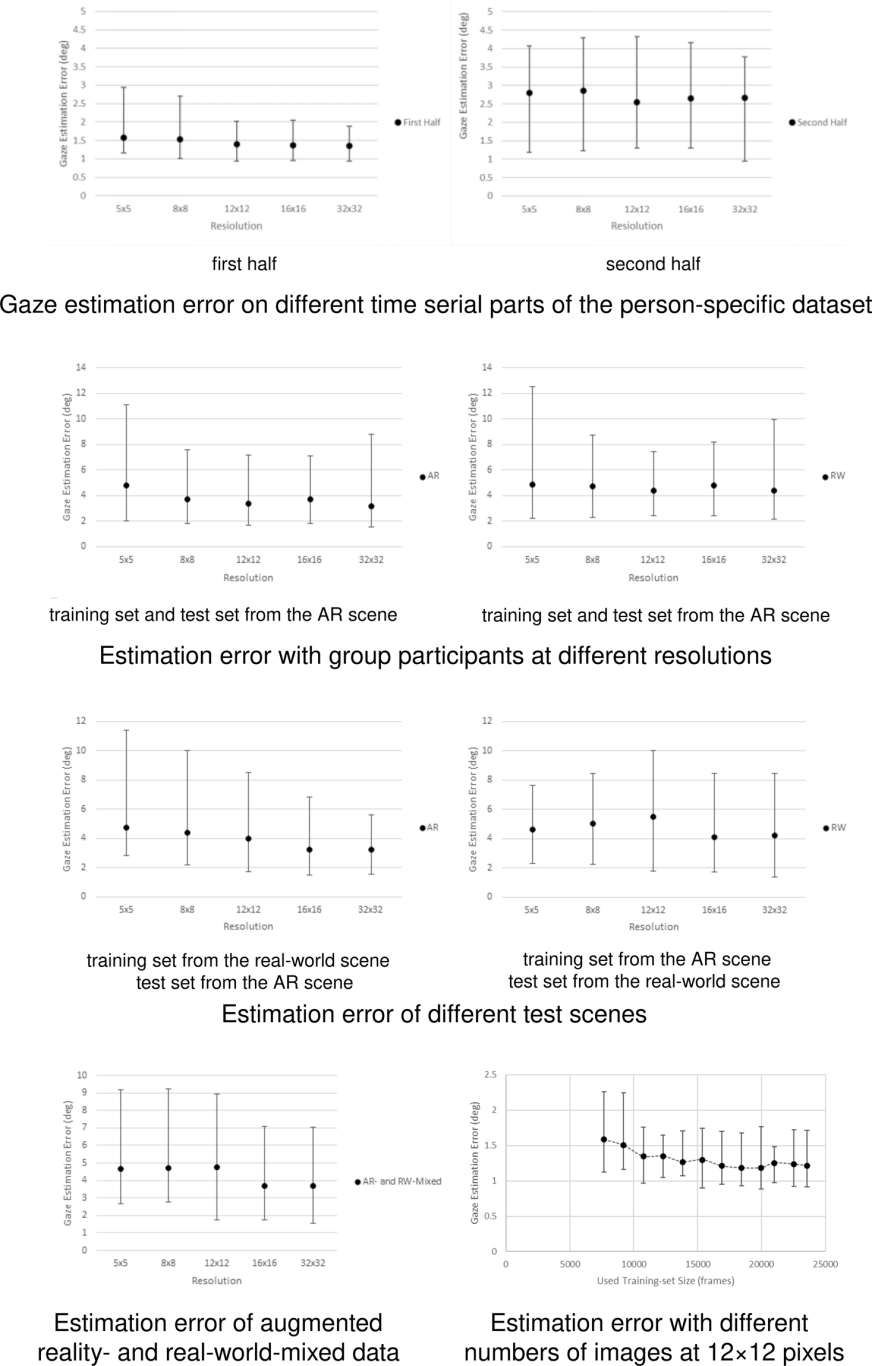
Result of estimation error with different dataset parameters.

We assessed how the number of images of individual participants affected eye gaze detection error (Fig. 8 forth row right). We extracted incremental numbers of images at 12 × 12 pixel resolution from each participant and tested the average eye gaze estimation error across the participants. The validation results with the fixed test set reported that the average estimation error decreased from 1.59 degrees (training data with 7,680 images) to 1.21 degrees (training data with 16,896 images) as the size of the training set increased. The results showed a noticeable decline in estimation error when sample sizes reached 16,896 images per participant. Given these results, it is not unreasonable to argue that the overall number of images in the dataset is sufficient for accurate eye tracking estimation and for the following validation.

#### features (angles) of the images in the dataset

The ARGaze dataset used eye images instead of face images, as face images had dynamic camera-eye spatial positions and the overall image quality was lower because of head movements, such as rotations. In contrast, eye images were captured through the statically mounted headset from an optimised angle of view (26.9 degrees horizontally and 13.0 degrees vertically). This reduced false detection of a variety of eye movements, such as squint, lift (i.e., looking upwards), droop (i.e., looking downwards), and blink. In addition, it provided geometric relationships of the eyes to support eye fixation prediction. We compared estimation results with monocular and binocular inputs (Fig. 7 third row) and found that the binocular images gained advantages with respect to estimation error. Compared with the monocular inputs, the average estimation error of the model is significantly lower when using binocular inputs. Taking the results of 12 × 12 pixels as an example, the average estimation error was 1.46 degrees with camera 1 and 1.35 degrees with camera 2, and the average estimation error was 1.10 degrees with both cameras. Statistical tests reported significant differences between the results with one camera and dual cameras (Mann–Whitney U test, p = 0.0008).

#### temporal stability of the dataset

Since the sequential eye gazes were affected by the preceding visual attention activities and retrieval practice effect, we examined possible fluctuations in eye image quality throughout the long eye tracking tasks. We tested the estimation error of the first and second half of each participant’s data across different resolutions (Fig. 8 first row). The results showed an increase in estimation error in the second half of the task data, but no significant differences were reported, which means that the ARGaze dataset has stable validity in the timing sequence (Mann–Whitney U test, p = 0.164).

#### generalisability of the dataset

Generalisability refers to how the dataset could effectively support other eye tracking applications, such as gaze estimation with individual participants and use in different groups of participants scenes. For individual participants, we trained the hybrid model with a training set and evaluated the estimation accuracy with the participant’s test set in the same scenario, as presented in the prior sections. For group participants, we divided the ARGaze dataset into two groups (N_1_ = 12, N_2_ = 13), trained the hybrid model with the dataset of one group (N_1_) and tested it with the data from the other group (N_2_). Both the training and test were performed several times with the same scenes at different resolutions (Fig. 8 second row). The lowest gaze estimation error at 32 × 32 pixels was 1.56 degrees in the AR scene and 2.16 degrees in the real-world scene. The mean gaze estimation error was 3.20 degrees (SD = 1.57) in the AR scene and 4.41 degrees (SD = 2.63) in the real-world scene.

Unlike the InvisibleEye dataset, which employed individual participant data for model training and evaluation and needed to record the training set before individual use, the ARGaze dataset supported effective eye gaze estimation with data from multiple nonoverlapping participants. Note that the ARGaze dataset was derived for multiuser calibration-free eye tracking and the InvisibleEye model was specialised for person-specific calibration-free eye tracking; thus, we did not compare multiuser-based estimation error with either the InvisibleEye or the hybrid model.

To validate the overall quality of the ARGaze dataset, we trained the hybrid model with the data from 15 tasks in augmented reality and real-world scenes and tested it with data from 11 similar tasks. Using the trained model, we calculated the mean error of eye gaze direction estimation per image in both augmented reality and real-world scenes with the same initial parameters at different epochs from 1 to 60 (Fig. 8 third row). The validation results with the data of non-trainset participants reported that the lowest estimation error at 32 × 32 pixels was 1.54 degrees in augmented reality scenes and 1.38 degrees in real-world scenes. The mean estimation error at 32 × 32 pixels was 3.22 degrees (SD = 1.16) in the augmented reality scene and 4.19 degrees (SD = 1.59) in the real-world scene.

To validate the generalisability of the ARGaze dataset across scenes, we trained the hybrid model with the data of a random group of participants in the augmented reality- and real-world-mixed scenes and tested it with the nonoverlapping data of the study participants. The results reported that the minimum error at 32 × 32 pixels was 1.56 degrees, and the average error was 3.70 degrees (Fig. 8 forth row).

#### (vi) overview of the ARGaze dataset

We compared the ARGaze dataset with other existing eye tracking datasets to further explain its overall effectiveness and generalisability. The results are summarised in Table 4. The effectiveness of the ARGaze dataset is confirmed by technical validation. First, it has 1,321,968 eye gaze images that were captured from 25 participants. The overall dataset size is larger than most existing datasets. To the best of our knowledge, the ARGaze dataset is the largest dataset that specialises in calibration-free eye tracking in real-world and augmented reality scenes. These images underwent strict technical validation to ensure overall image quality. Despite the small image resolutions of the proposed dataset, the 32 × 32 pixel resolution is proven to be effective for training and testing the calibration-free eye tracking model. The small resolution images also allow quick iteration of model development. In addition, we supply the resampled eye gaze images at 512 × 512 pixel in.mp4 video sequences.

**Table 4:** Comparisons of the current eye tracking datasets.

	Participants	On-Screen Gaze Targets	Images	Resolution	Scenes	Single User Angle Error (Lowest)	Multiuser Angle Error (Average)
ARGaze	25	continuous	1,321,968	32 × 32	AR, real world	0.78(AR) & 0.90(RW)	3.70(mixed)
MPIIGaze^18^	15	continuous	213,659		real world		4.56
Eyediap^8^	16	continuous	videos	640 × 480	real world		5.84
ShanghaiTechGaze + ^19^	218	continuous	165,231	1920 × 1080	real world		4.8
UT Multiview^20^	50	discrete	64,000		real world		4.41(left eye) & 4.24(right eye)
InvisibleEye^21^	17	discrete	28,000	250 × 250	real world	1.79	
NVGaze^22^	30	continuous	2,500,000	480 × 640	VR, AR	0.84	2.06
Stephan *et al.*^23^	152	continuous	356,649	400 × 640	VR		
Magic Eyes^24^	587	continuous	880,000	480 × 640	MR		4.22
TEyeD^25^	132	continuous	29,867,973	480 × 640, 288 × 384, 240 × 320, 360 × 640	VR, AR, real world		

Second, the ARGaze dataset is diverse in terms of participant backgrounds (i.e., gender-balanced), eye gaze scenes (i.e., real-world and augmented reality scenes), image sources (i.e., left eye, right eye, and world view), and eye features (i.e., eye colours and shapes from subjects of different ethnicities).

Third, the ARGaze dataset outperforms the existing datasets in terms of calibration-free eye gaze estimation accuracy in both real-world and augmented reality scenes, and its accuracy is higher in real-world scenes. With replicable camera hardware configurations and machine learning models, the dataset develops device-dependent eye tracking systems to eliminate user-dependent calibrations. In contrast with the NVGaze dataset, which achieved higher accuracy in multiuser eye gaze direction estimation in respective scenes of virtual reality and augmented reality (see details in Table 4), the ARGaze dataset combines eye gaze images of real-world and augmented reality scenes to enable scene-independent calibration-free eye tracking.

The generalisability of the ARGaze dataset is threefold. First, with leading scale and diversity, the ARGaze dataset can be employed as a conventional dataset to develop eye gaze estimation methods for eye tracking devices with other configurations. Second, the ARGaze dataset is generalised for real-world and/or augmented reality scenes with high estimation accuracy, and also has good compatibility with mainstream machine learning models that are openly available to researchers. Third, the ARGaze dataset is presented together with the specific hardware configurations (e.g., camera positions and field of view) and machine learning models. With the replicable specifications provided in technical validation, researchers can develop eye tracking devices that no longer require calibrations regardless of eye tracking users and scenes. Generally, it is technically and economically efficient to develop a calibration-free eye tracking device using the ARGaze dataset and its related configurations and models, as the conventional construction of eye gaze estimation methods relies heavily on users and scenes.

## Usage Notes

We make the dataset public for researchers to test and optimise their eye-tracking algorithms. We expect both user-dependent and user-independent models to be designed and achieve high accuracy with the dataset. We described detailed processes about data cleaning, ground truth, and model training so that the dataset and related models can be used in other generalising eye-tracking projects.

## Data Availability

Software applications used in the study are based on public open sources, and the codes used in this study are available at https://github.com/xdc-lab/ARGazeCodes. All the codes used in the preceding data processing and technical validation are available at https://github.com/xdc-lab/ARGazeCodes.
