# Author Correction: A dataset of eye gaze images for calibration-free eye tracking augmented reality headset

**DOI:** 10.1038/s41597-023-02201-3

**Published:** 2023-05-16

**Authors:** Zihan Yan, Yue Wu, Yifei Shan, Wenqian Chen, Xiangdong Li

**Affiliations:** College of Computer Science and Technology, Zhejiang University, Hangzhou, 310027 P. R. China

**Keywords:** Visual system, Saccades, Biomedical engineering

Correction to: *Scientific Data* 10.1038/s41597-022-01200-0, published online 29 March 2022.

Equations 11 and 12 in the Technical Validation section were incomplete in the original version. The correct versions are as follows:

11 $${{\rm{L}}}_{{\rm{Training}}}=\frac{1}{{\rm{N}}}\sum {(| | {\rm{Y}}-{{\rm{Y}}}_{0}| {| }_{2})}^{2}=\frac{1}{{\rm{N}}}\sum ({({\rm{x}}-{{\rm{x}}}_{{\rm{0}}})}^{2}+{(<?RemoveMO2?>{\rm{y}}-{{\rm{y}}}_{{\rm{0}}})}^{2}){\rm{,}}$$

12 $${{\rm{L}}}_{{\rm{Validating}}}=\frac{1}{{\rm{N}}}\sum {| | {\rm{Y}}-{{\rm{Y}}}_{0}| | }_{2}=\frac{1}{{\rm{N}}}\sum \sqrt{{({\rm{x}}-{{\rm{x}}}_{{\rm{0}}})}^{2}+{({\rm{y}}-{{\rm{y}}}_{{\rm{0}}})}^{2}}{\rm{,}}$$

These have been corrected in the pdf and HTML versions of the paper.

